# The Risk of Mistaking Intestinal Lanthanum Carbonate for Intestinal Bleeding on CT

**DOI:** 10.5334/jbr-btr.852

**Published:** 2015-09-15

**Authors:** D. Salerno, P. E. Colin, C. Ernotte, B. Dubois, P. Meunier

**Affiliations:** 1 Department of Radiology, Centre Hospitalier Universitaire de Liège, Rue Pirette, 4, B-4602 Cheratte, Belgium; 2 Department of Nephrology/Dialysis, Centre Hospitalier Universitaire de Liège, Sart Tilman, B-4000 Liège, Belgium

Dear Editor,

A 65 year-old male was referred to our department of radiology for investigation of rectorrhagia. The patient had a history of hypercholesterolemia, arterial hypertension, type II diabetes, intestinal polyps, diverticulosis, and monoclonal gammopathy leading to chronic renal failure requiring recurrent hemodialysis. During the previous week, the patient had been suffering from severe pulmonary infection accompanied by septic shock with hepatitis. Gastroscopy and colonoscopy were unable to find the cause of the bleeding because of a lack of compliance to the preparation diet. A computed tomography was requested to locate the source of the bleeding.

The pre-contrast acquisition showed a number of spontaneously hyper-dense elements throughout the whole intestinal tract, inside the bowel lumen, from the stomach to the rectum exhibiting very sharp edges (better seen on the scout view). The opacities determined marked attenuation of X-rays with densities around 2500 Hounsfield Units (HU) and significant beam-hardening artifacts (Fig. 1).

**Figure 1:**
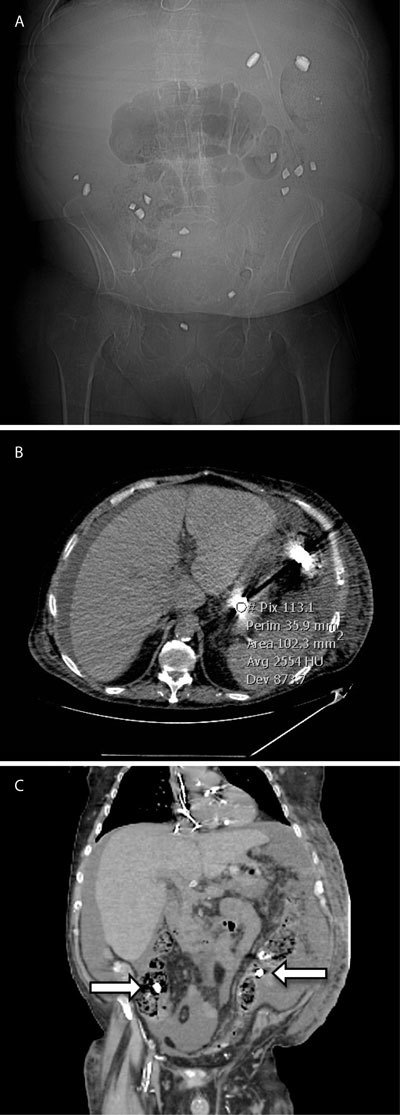
A. Scout examination (prior to the CT acquisition) revealing multiples sharp-edged high-density opacities of random distribution inside the abdomen. B, C.: Computed tomography showing the high density (2500 HU) lanthanum carbonate tablet (arrows) in the stomach and the colon, inducing beam-hardening artifacts.

It was first thought that the patient had ingested some kind of foreign body (as suggested by the appearance on the scout examination) that could explain the intestinal bleeding, which he denied. There was also no history of a recent administration of oral contrast. No other relevant cause of bleeding per rectum was found on the CT-scan images. The patient stopped bleeding in the meantime without further intervention. Clinicians from the hemodialysis unit pointed out that the patient had been receiving oral lanthanum carbonate for the last couple of days (1000 mg per day).

Lanthanum carbonate is used in the treatment of hyperphosphatemia, often occurring in patients with chronic renal failure. Binding the alimentary phosphate, it reduces the absorption of phosphate in the small bowel. Lanthanum is a heavy metallic element of the third group of the periodic table (the lanthanides), which also includes barium and gadolinium. Its nature itself thereby explains why tablets of lanthanum carbonate exhibit such marked X-rays attenuation. As it has been reported, well-chewed pills can opacify the intestinal lumen, mimicking oral contrast medium. When not or partially chewed, the tablets will appear as small opacities of high density with beam-hardening artifacts, such as in our report.

Whilst many nephrologists know that lanthanum carbonate is a radio-opaque substance, few radiologists do and should therefore pay more attention to the patient’s food habits as well as the drugs he is receiving to avoid misinterpretations of the radiological images.

## Competing Interests

The authors declare that they have no competing interests.
